# Urethral Calculi

**Published:** 1902-02

**Authors:** J. Hugo Reed

**Affiliations:** Guelph, Ontario


### REPORTS OF CASES.

### URETHRAL CALCULI.

By J. Hugo Reed, V.S.,
GUELPH, ONTARIO.

On October 14th I was called to see a grocer's delivery horse whose driver said he could not defecate. I had him brought to my infirmary. He was evidently endeavoring to defecate. Pulse about 45 and normal in volume; temperature normal; penis slightly protruded, but no apparent efforts were made to urinate. I treated for impaction of the colon with paralysis of the rectum. The next morning, while examining the rectum, I found the bladder distended, and in trying to pass a catheter met with an obstruction about five inches above the meatus. Manipulation located a calculus, but failed to move it. I cut down and removed it. It had a rough surface, was nearly round, measured three-quarters of an inch in diameter, and weighed two drachms. Excessive swelling took place at once, sufficient to interfere with suturing. I succeeded in suturing with cauterized catgut. The swelling continued, and I scarified the swollen parts, bathed with hot water, and applied refrigerants. The next morning the swelling was considerably reduced. I passed a catheter twice daily and sent him home on the 28th. On November 6th he was brought back, acting much the same as at first. In again passing the catheter I located another calculus just above the ischial arch. This was also immovable by manipulation, and I cut down and removed it. It was of the same nature as the first, but was irregular in form, measured $1\frac{3}{8}$ inches in the longest diameter, and weighed 6 ounces. I stretched the urethra, but left the external tissues open. I again passed the catheter regularly and treated the wound with antiseptics. No urine escaped through the wound. On the 11th I was forced to leave home for four days, leaving the case in charge of an assistant. On my return I found urine escaping in considerable quantities from the wound. Taking hold of the ends of the sutures, they pulled away easily, bringing with them what appeared to be a portion of the urethra, which had sloughed. I then filled the wound with equal parts of boric acid and iodoform, and stitched it closely. After a few days a little urine passed through, but this gradually became less, and I sent him home on the 22d. In about four days the escape of urine had entirely ceased. I removed the stitches, and he has been working regularly since. He does not urinate quite as freely as normal, but suffers no inconvenience.

